# Supplementary material for: Design principles to tailor Hsp104 therapeutics
Source: bioRxiv. 2024 Apr 28:2024.04.26.591398. Preprint. [Version 1] doi: 10.1101/2024.04.26.591398 (PMC11071516; doi:10.1101/2024.04.26.591398)
Supplement: 2 [file NIHPP2024.04.26.591398V1-supplement-1.pdf]

bioRxiv preprint doi: https://doi.org/10.1101/2024.04.26.591398; this version posted April 28, 2024. The copyright holder for this preprint (which was not certified by peer review) is the author/funder, who has granted bioRxiv a license to display the preprint in perpetuity. It is made available under aCC-BY 4.0 International license.

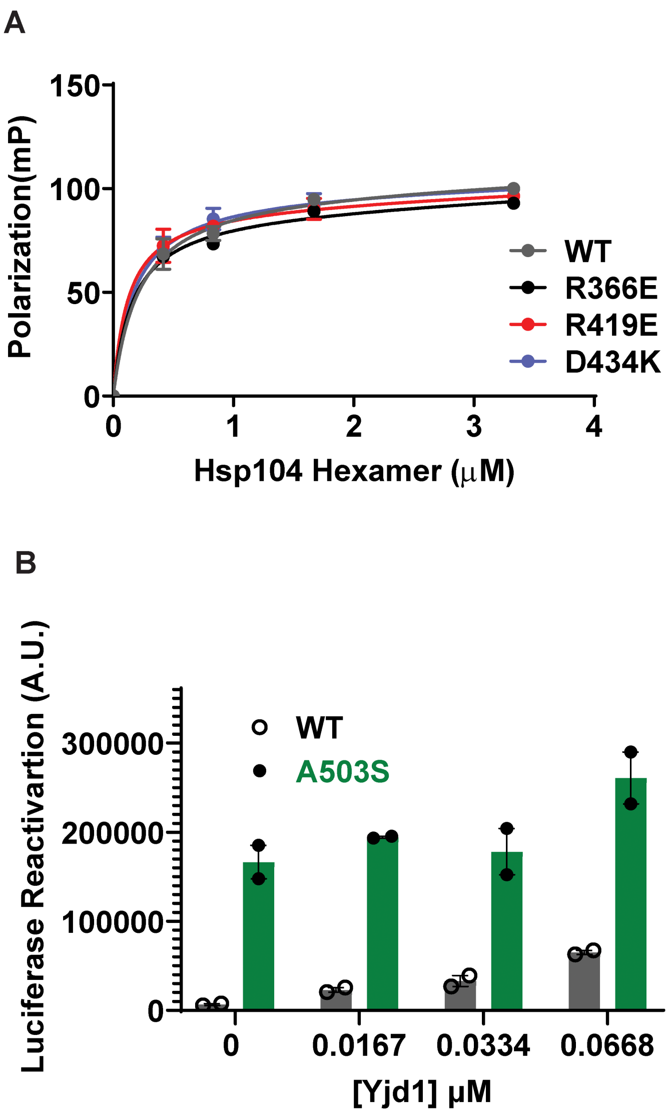

Figure S1. Hsp104 variants bind a model, disordered substrate, β-casein, with the same affinity as Hsp104 (**A**) FITC-casein (30nM) was incubated with the indicated concentration of Hsp104 (x-axis) in the presence of ATPγS (2mM). Binding was assessed by fluorescence polarization. Values represent means±SEM (N=2). The data were fitted using a one-site binding curve in Graphpad, and the apparent K_D of the Hsp104 variants tested are similar to WT Hsp104 (0.2±0.1μM). (**B**) Bar graph of the data presented in Figure 2E for luciferase disaggregation and reactivation by Hsp104 or Hsp104^{A503S} (1μM, monomeric), plus Ssa1 (0.167μM) and the three lowest Ydj1 concentrations or in the absence of Ydj1. Bars represent means±SEM (N=2); each data point represents an independent replicate.

Related to Figure 1 and 2.

bioRxiv preprint doi: https://doi.org/10.1101/2024.04.26.591398; this version posted April 28, 2024. The copyright holder for this preprint (which was not certified by peer review) is the author/funder, who has granted bioRxiv a license to display the preprint in perpetuity. It is made available under aCC-BY 4.0 International license.

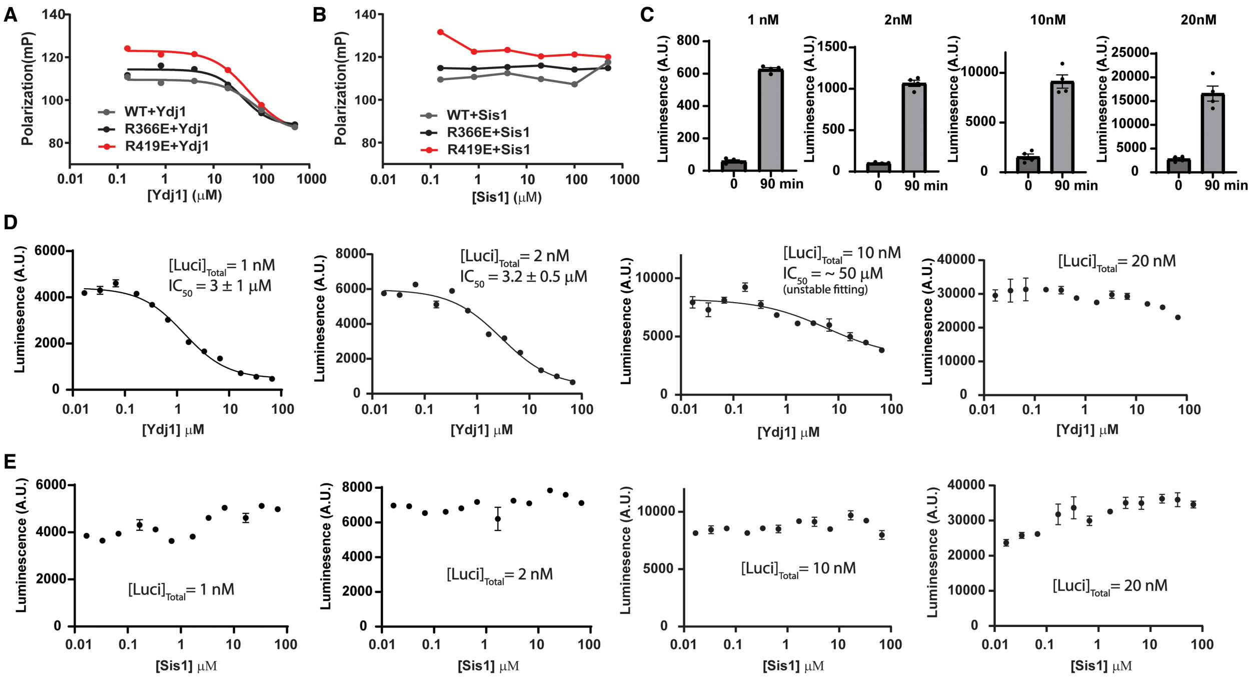

Figure S2. Ydj1 but not Sis1 can dissociate substrate from Hsp104 and inhibit the spontaneous refolding of unfolded luciferase. (A, B) Fluorescence polarization experiments measuring substrate binding competition between Hsp104 and Hsp40. Hsp104 (5μM hexameric) and the model substrate, FITC-casein (30 nM), were incubated with ATPγS (2mM) for 30min. The complex was then titrated with Ydj1 (A) or Sis1(B) at the indicated concentrations (x-axis, log scale) in the presence of ATPγS (2mM). Fluorescence polarization of FITC-casein (y-axis) was measured. Results from a representative experiment are shown. (**C**) Spontaneous refolding of soluble unfolded luciferase in buffer was measured at time of unfolding (0 min) and after 90 min. Luciferase (10μM) in 6M urea was incubated on ice for 5min and then diluted into solutions to a final concentration of 1, 2, 10 or 20nM as indicated in the figure. Luciferase activity was measured right after the unfolding reaction or after 90min in buffer. Bars represent means±SEM (N=4), each replicate is shown as a dot. (**D, E**) Luciferase (10μM) in 6M urea was incubated on ice for 5 min and then diluted into solutions containing various concentrations of Ydj1 (panel D x-axis, log scale) or Sis1 (panel E x-axis, log scale) to a final concentration of 1, 2, 10 or 20nM as indicated. Luciferase activity was measured after 90min. Values represent means±SEM (N=2). The IC_{50} of Ydj1 inhibition was determined using the dose-dependent fitting model for absolute IC_{50}.

Related to Figure 2 and Table S1.

bioRxiv preprint doi: https://doi.org/10.1101/2024.04.26.591398; this version posted April 28, 2024. The copyright holder for this preprint (which was not certified by peer review) is the author/funder, who has granted bioRxiv a license to display the preprint in perpetuity. It is made available under aCC-BY 4.0 International license.

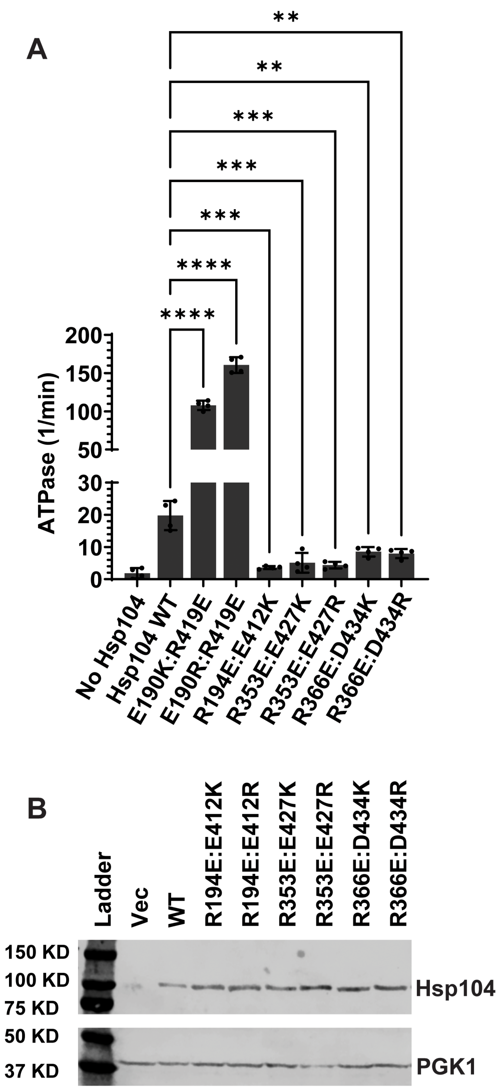

Figure S3. Rebuilding of the NBD1:MD salt bridges alters the ATPase activity of Hsp104. (**A**) ATPase activity of the indicated Hsp104 variants (0.25μM, monomeric) in ATP (1mM) after 5min at 25°C. Bars represent means±SEM (N=4), individual replicates are shown as dots. Dunnett's multiple comparisons were performed to compare the ATP hydrolysis rate of NBD1-MD variants to WT. **** P≤0.0001, ***P≤0.001, **P≤0.01. (**B**) Western blots to evaluate Hsp104 expression level of yeast in the thermotolerance assay (Figure 3B). Hsp104 variants were expressed for 30min at 37°C in Δ*hsp104* yeast. Yeast were then lysed, and the lysates were processed for Western blot. PGK1 serves as a loading control.

Related to Figure 3.

bioRxiv preprint doi: https://doi.org/10.1101/2024.04.26.591398; this version posted April 28, 2024. The copyright holder for this preprint (which was not certified by peer review) is the author/funder, who has granted bioRxiv a license to display the preprint in perpetuity. It is made available under aCC-BY 4.0 International license.

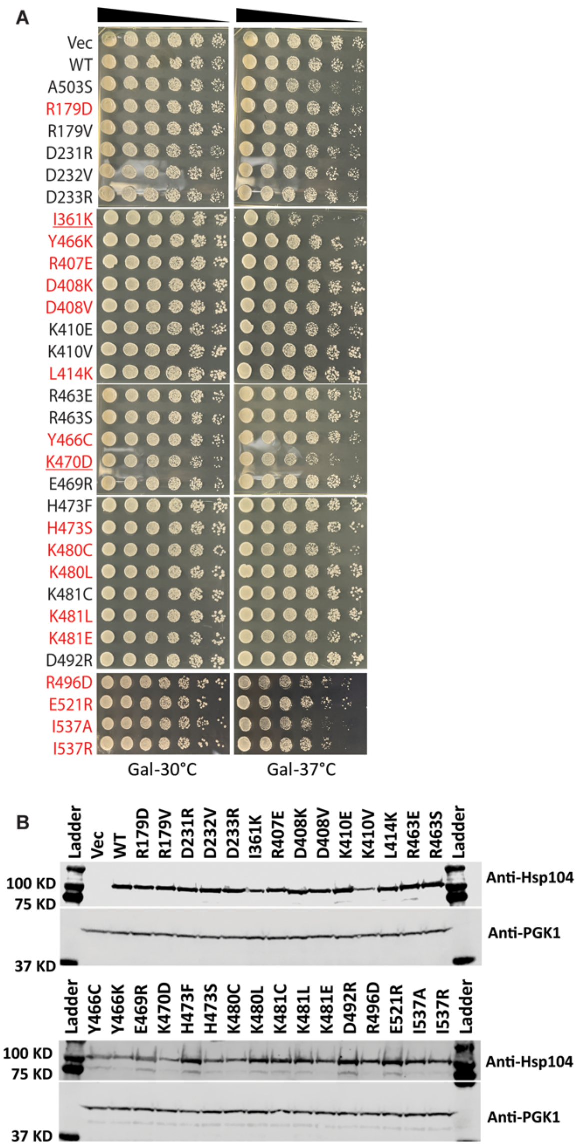

Figure S4. Hsp104 variant off-target toxicity and expression level in yeast. (**A**) The off-target toxicity of Hsp104 variants that perturb the intraprotomer NBD1:MD contacts of the ADP state is evaluated at 37°C using yeast spotting assay. *Δhsp104* yeast were transformed with galactose-inducible Hsp104 variants or an empty vector, WT Hs104 or Hsp104^{A503S} serve as controls. The yeast were spotted onto galactose (induction on) media in a five-fold serial dilution and incubated at 30°C (left) or 37°C (right). The potentiated variants revealed in Figure 4 are highlighted in red, and the toxic variants are underlined. (**B**) Western blots were performed to evaluate Hsp104 expression. *Δhsp104* yeast from Figure 4 harboring the indicated Hsp104 variants or empty vector control were induced in galactose media for 5 hours at 30°C. Yeast were lysed and the lysates were visualized via Western blot. PGK1 serves as a loading control.

Related to Figure 4.

bioRxiv preprint doi: https://doi.org/10.1101/2024.04.26.591398; this version posted April 28, 2024. The copyright holder for this preprint (which was not certified by peer review) is the author/funder, who has granted bioRxiv a license to display the preprint in perpetuity. It is made available under aCC-BY 4.0 International license.

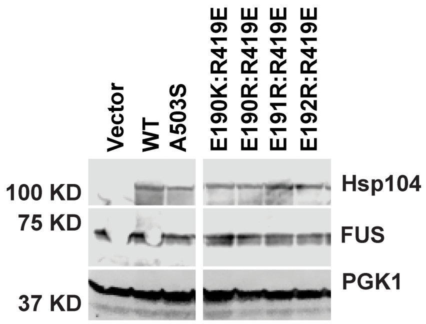

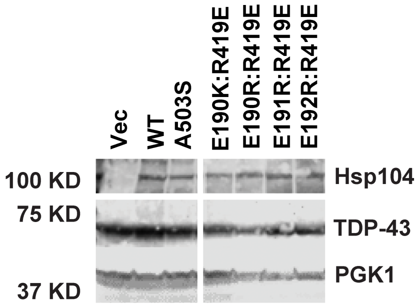

Figure S5. Western blots confirm Hsp104 variants and disease proteins are expressed at similar levels. Integrated Δ*hsp104* yeast strains from Figure 6C (left) and 6D (right) were induced for 5 hours in galactose media. Yeast were lysed and processed for western blot. PGK1 serves as a loading control.

Related to Figure 6.

bioRxiv preprint doi: https://doi.org/10.1101/2024.04.26.591398; this version posted April 28, 2024. The copyright holder for this preprint (which was not certified by peer review) is the author/funder, who has granted bioRxiv a license to display the preprint in perpetuity. It is made available under aCC-BY 4.0 International license.

	**1. Ssa1 EC_{50}** (Figure 2B)	**2. Sis1 EC_{50}** (Figure 2D, F)	**3. Ydj1 EC_{50}** (Figure 2C, E)	**4. Ydj1 IC_{50}** (Figure 2C, E)	**5. Ydj1 IC_{50}** (Figure S2A)
**Hsp104**	~5μM	~2μM	~0.4μM	~14μM	~40μM
**Hsp104^{R366E}**	~6μM	~1.9μM	~0.047μM	~0.63μM	~20μM
**Hsp104^{R419E}**	~5μM	~3μM	~0.08μM	~1.3μM	~30μM
**Hsp104^{A503S}**	ND	~2μM	~0.21μM	~6μM	ND

Table S1. Summary of EC_{50} and IC_{50} values. From left to right: 1. EC_{50} of Ssa1 for stimulation of luciferase disaggregation and reactivation by Hsp104 variants in the absence of Hsp40 (Figure 2B). 2. EC_{50} of Sis1 for stimulation of luciferase disaggregation and reactivation by Hsp104 variants in the presence of Ssa1 (Figure 2D, F). 3. EC_{50} of Ydj1 for stimulation of luciferase disaggregation and reactivation by Hsp104 variants in the presence of Ssa1 (Figure 2C, E). 4. IC_{50} of Ydj1 for stimulation of luciferase disaggregation and reactivation by Hsp104 variants in the presence of Ssa1 (Figure 2C, E). 5. IC_{50} of Ydj1 for dissociating β-casein from Hsp104 variants (Figure S2A).
